# HDAC2 promotes the EMT of colorectal cancer cells and via the modular scaffold function of ENSG00000274093.1

**DOI:** 10.1111/jcmm.16186

**Published:** 2020-12-15

**Authors:** Zhi‐Peng Qi, Ayimukedisi Yalikong, Jia‐Wei Zhang, Shi‐Lun Cai, Bing Li, Sun Di, Zhen‑Tao Lv, En‐Pan Xu, Yun‐Shi Zhong, Ping‐Hong Zhou

**Affiliations:** ^1^ Endoscopy Center Zhongshan Hospital of Fudan University Shanghai China; ^2^ Endoscopy Research Institute of Fudan University Shanghai China; ^3^ Department of internal medicine of Xuhui Hospital Affiliated Zhongshan Hospital Fudan University Shanghai China

**Keywords:** colorectal cancer, epithelial‐mesenchymal transition, histone deacetylase 2, long non‐coding RNAs

## Abstract

Histone deacetylase 2 (HDAC2), a member of the Histone deacetylase family, plays a vital role in various carcinomas. In this study, we identified that HDAC2 expression levels are associated with liver metastasis, higher T stages and poor prognosis in colorectal cancer. HDAC2 down‐regulation via lentivirus‐mediated expression of HDAC2‐targeting shRNA reduced the in vitro migration and invasion ability of HCT116 cell as well as their liver metastasis in nude mouse xenografts. Mechanistically, HDAC2 promotes epithelial‐mesenchymal transition (EMT) in colorectal cancer cells by combining HDAC1 with EZH2 (a key histone methyltransferase), possibly through the modular scaffold function of a new lncRNA, ENSG00000274093.1. HDAC2 thus appears to promote CRC cell migration and invasion through binding HDAC1 and EZH2 via ENSG00000274093.1.

## 1 INTRODUCTION

Colorectal cancer (CRC) is the third most commonly diagnosed cancer, with an estimated 1.1 million cases and 551 269 deaths in 2018,^1^ and liver metastasis is the main cause of death in patients with colorectal cancer.^2^ Therefore, to improve the diagnosis and treatment of colorectal cancer, researching the liver metastasis of CRC is necessary. Recent studies revealed histone acetylation, one of the most extensively studied histone modifications, is regulated by the histone deacetylases (HDACs), and play a pivotal role in cancer development and progression.^3^, ^4^ Given the fact that the activity of HDACs is dysregulated in many types of cancer, thus HDACs have been considered as therapeutic targets for the treatment of cancers.

HDAC2 has reportedly overexpressed in liver, cervical and lung cancers.^5^, ^6^, ^7^ There is also a high expression of HDAC2 in the carcinogenesis of colorectal cancer. Anne et al analysed 254 cases of colorectal cancer and 50 cases of normal colorectal mucosa. The results showed that there was an increase of HDAC2 and an abnormal level of H3K56 acetylation in colorectal cancer tissues.^8^ Zhu and other studies have shown that HDAC2 plays an important role in the development of colorectal cancer and can be used as a potential therapeutic target.^9^ However, at present, there is still a lack of research on the role of HDAC2 in colorectal liver metastasis and its regulatory mechanism.

LncRNA is a class of non‐coding transcripts greater than 200 nucleotides in length. Recent studies have revealed that lncRNAs may affect development, metabolism and other aspects of cancer.^10^, ^11^ The role of lncRNAs and the underlying mechanisms in CRC also has been reported before.^12^, ^13^, ^14^, ^15^ However, more specific mechanisms of lncRNAs in CRC need to be further teased out.

In the present study, we found that HDAC2 was highly expressed in colorectal cancer compared to adjacent normal mucosa. Clinicopathologic data indicated that higher HDAC2 expression correlated with poor overall survival (OS) and was associated with liver metastasis and higher T stages. Besides, HDAC2 knockdown prevented cell migration and invasion. This process requires a novel LncRNA, ENSG00000274093.1, which binds to HDAC2 and may act as a modular scaffold for the HDAC1/HDAC2 and EZH2 complexes, thereby altering EMT in colorectal cancer cells.

## 2 MATERIALS AND METHODS

### 2.1 Patient specimens

Tumours and the adjacent colorectal tissues were obtained from patients with colorectal cancer who underwent surgery at Zhongshan Hospital, Fudan University, from June 2016 to July 2016. The study protocol was approved by the ethics committee of Zhongshan Hospital, Fudan University. Written informed consent was obtained from all participants in this study. All the research was carried out in accordance with the provisions of the Declaration of Helsinki. None of these patients had received radiotherapy or chemotherapy before surgery. The percentage of tumour cellularity in the tissue section was at least 70% as determined by a pathologist.

### 2.2 Immunohistochemistry

HDAC2 expression was measured in tumour samples using a Histostain®‐Plus 3rd Gen IHC Detection Kit (Invitrogen, USA #85‐9073) following the manufacturer's instructions. The tissue microarray (TMA) slide was dried overnight at 37°C, deparaffinized in xylene, rehydrated through graded alcohol, immersed in 3% hydrogen peroxide for 20 minutes to block endogenous peroxidase activity and then antigen‐retrieved by microwave heating in 0.01 M sodium citrate buffer (pH = 6.0) at 100°C for 30 minutes. Subsequently, slides were pre‐incubated with 10% normal goat serum at room temperature (RT) for 30 minutes to reduce the non‐specific reaction. The primary rabbit anti‐HDAC2 polyclonal antibody (ab41587, Abcam) was diluted (1:50) in phosphate buffered saline (PBS) with 3% bovine serum albumin (BSA) and applied overnight in a humidity chamber at 4°C. Slides were incubated with a polymer peroxidase‐labelled secondary antibody for 30 minutes at RT and then stained with DAB. Finally, sections were counterstained with haematoxylin.

The expression of HDAC2 was determined by the positive tumour cells proportion and the average intensity of positive tumour cells in the tumour tissues. Four levels were defined: no staining = ‐, weak staining = +, moderate staining = ++ and strong staining = +++. The final score was classified as low or high expression using the median value as the cut‐off.

### 2.3 Cell culture

The human colorectal cancer cell lines HCT116 and LoVo were obtained was maintained in DMEM medium supplemented with 10% foetal bovine serum and 100 units/ml penicillin‐streptomycin (Invitrogen, USA) at 37°C in 5% CO_2_.

### 2.4 Co‐immunoprecipitation

Co‐immunoprecipitation was performed as described previously. In brief, both input and IP samples were analysed by Western blot using various antibodies at the indicated dilutions: anti‐HDAC1 antibody (1:1,000; Cell Signal Technology), anti‐EZH2 antibody (1:1,000; Cell Signal Technology) and normal rabbit IgG.

### 2.5 RNA immunoprecipitation

RNA immunoprecipitation (RIP) experiments were performed using the Megna RIP RNA‐binding Protein Immunoprecipitation Kit (Millipore). The anti‐HDAC2 antibody used for RIP was purchased from CST (Cell Signaling Technology). The co‐precipitated RNAs were detected by reverse transcription PCR. To demonstrate that the detected RNA signals specifically bind to HDAC2, total RNA (input controls) and normal rabbit IgG controls were simultaneously assayed.

RNA Analysis, Extraction and Quantitative Real‐Time PCR:The RNA expression levels were measured using a real‐time quantitative PCR system. Total RNA was extracted by TRIzol reagent (Invitrogen), and 1 μg of total RNA was reverse‐transcribed using the PrimeScriptP RT Reagent Kit (Perfect Real Time; Takara). The amplified transcript level of each specific gene was normalized to that of 18S of GAPDH. The primers were provided by Shenggong Company (Table S1).

### 2.6 RNA pull‐down assay

Cell nuclear lysates were incubated with biotinylated RNA and streptavidin beads for RNA pull‐down incubation. Then, beads were collected by centrifugation. RNA‐associated proteins were eluted and resolved by SDS‐PAGE followed by silver staining (Bio‐Rad).

### 2.7 Fluorescence in situ hybridization (FISH) assay

Cells grown on coverslips were fixed with 4% paraformaldehyde at 25°C for 15 minutes, washed three times with PBS and treated with 0.5% Triton X‐100 at room temperature for 10 minutes. The samples were dehydrated in 3% H_2_O_2_ and air‐dried. After probe hybridization solution was added, the samples were mounted, denatured at 88°C for 5 minutes, and hybridized in a humid and dark environment at 37°C for 16‐72 hours with FITC‐labelled LncRNA ENSG00000274093.1 probe (Exon Biotechnology Inc, Shenggong, China). The samples were washed three times with a preheated (43°C) solution consisting of 50% formamide and 23 saline sodium citrate (SSC) and then washed twice with 23 SSC (37°C). After the samples were counterstained with 40,6‐ diamidino‐2‐phenylindole, they were mounted with fluorescence mounting medium and imaged with a microscope.

### 2.8 Transwell assay

Transwell assays were performed using 24‐well transwells (8‐μm pore size; Millipore) precoated with Matrigel (BD Biosciences). Cells at logarithmic phase were transfected with lentivirus‐mediated knockdown of HDAC2, and knockdown or overexpression of ENSG00000274093.1 or control HCT116 (Genechem, Shanghai). Transfected cells were then harvested, and 1 × 10^5^ cells were seeded in serum‐free medium into the upper chamber, whereas medium supplemented with 20% FBS was applied to the lower chamber as a chemoattractant. After 48 hours of incubation, the migrated cells at the bottom surface of the filter were fixed, stained and counted.

In *vivo* tumorigenicity assay: All animal experiments were performed in accordance with NIH guidelines for the use of experimental animals. Male Nu/Nu mice, obtained from the Shanghai Laboratory Animal Center (SLAC), were injected left forelimb subcutaneously with 8 × 10^6^ stable monoclonal HCT116 cells infected with lentivirus‐mediated knockdown of HDAC2, and knockdown or overexpression of ENSG00000274093.1 to establish a CRC xenograft model. Eighteen days after injection, mice were optical in vivo imaging and sacrificed; then, IHC was conducted.

### 2.9 Statistical analysis

Comparisons between groups were performed using paired Student's t tests, and differences were considered statistically significant at *P* < 0.05. Data were presented as the mean ± SD of three independent experiments.

## 3 RESULT

### 3.1 HDAC2 was highly expressed in colorectal cancer tumour tissues and was associated with poor overall survival

**FIGURE 1:**
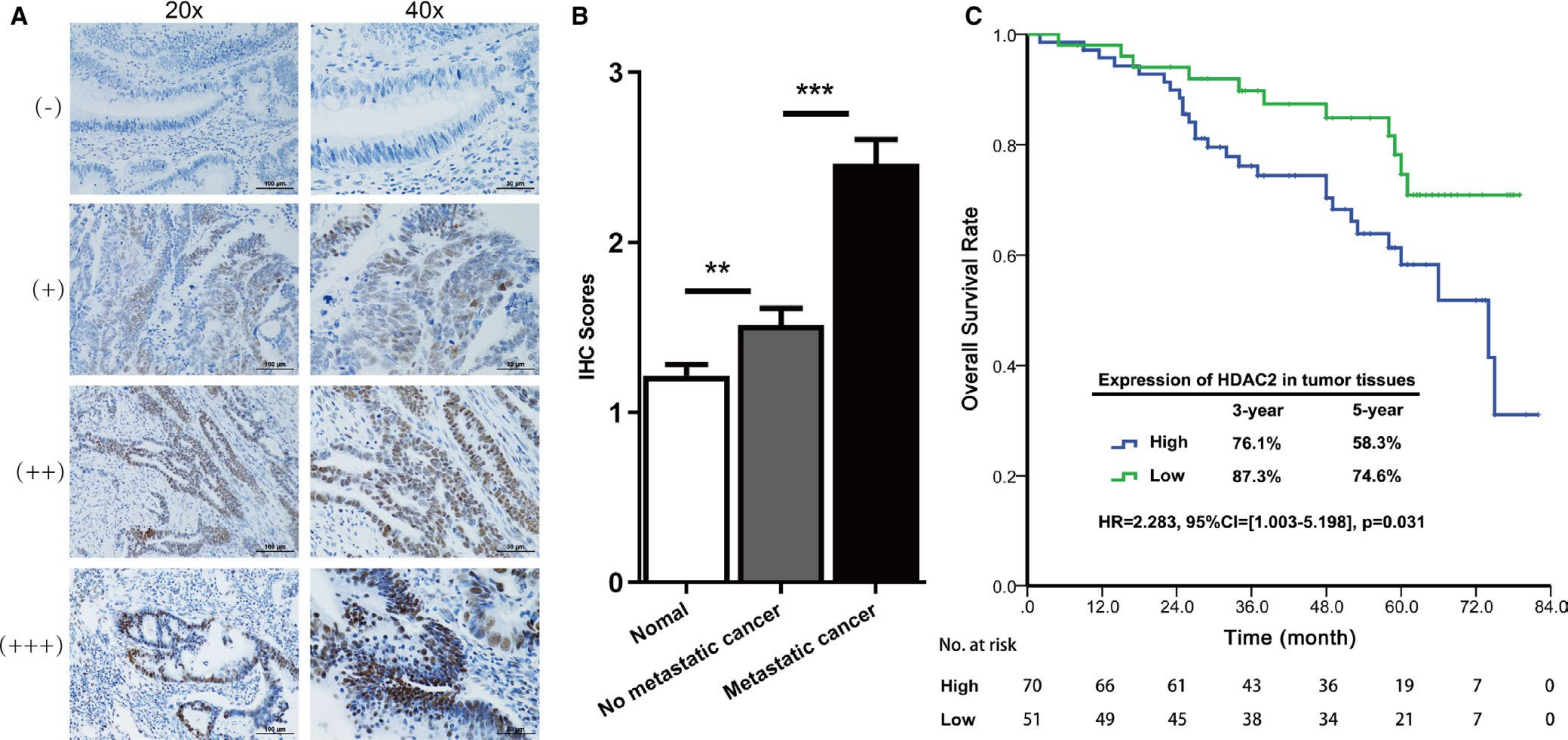
HDAC2 was highly expressed in colorectal cancer tumour tissues and was associated with poor overall survival. (A) Representative images of HDAC2 IHC staining intensity: (−) no staining; (+) weak staining; (++) moderate staining; (+++) strong staining. (B) HDAC2 IHC staining score was significantly higher in tumour tissues than in normal tissues. **<0.01; ***<0.001. (C) Kaplan‐Meier survival curve with HDAC2 expression in tumour tissues for CRC patients after primary tumour resection. HR: hazard ratio; CI: confidence interval; *P* value: log‐rank test

HDAC2 protein expression in the primary tumour tissue samples from 121 patients was detected by immunohistochemistry (IHC). Representative images (Figure 1A) show four different typical HDAC2 expression patterns. HDAC2 IHC scores in tumours with liver metastasis were significantly higher than those in tumours without liver metastasis (*P* < 0.01) and normal tissues (*P* < 0.001) (Figure 1B), suggesting that HDAC2 accumulated in CRC patients, especially in ones with liver metastasis.

For the IHC score, the median value was defined as the cut‐off between the HDAC2 high‐ and low‐expression subgroups. A total of 70 samples exhibited high expression, and 51 exhibited low expression. High HDAC2 expression was associated with primary pT stage 3/4 (*P* = .023) and liver metastasis (*P* < 0.001) (Table 1).

**TABLE 1:** Relationships between HDAC2 expression in tumour tissues and patient/tumour clinicopathologic characteristics

	High (%) n = 70	Low (%) n = 51	*P* value
Sex			
Male	53 (75.7%)	36 (70.6%)	.528
Female	17 (24.3%)	15 (29.4%)	
Age			
≤60	29 (41.4%)	28 (54.9%)	.143
>60	41 (58.6%)	23 (45.1%)	
Primary tumour site			
Right‐sided	21 (30.0%)	19 (37.3%)	.222
Left‐sided	27 (38.6%)	12 (23.5%)	
Rectum	22 (31.4%)	20 (39.2%)	
Primary tumour size			
≤4 cm	43 (61.4%)	32 (62.7%)	.883
>4 cm	27 (38.6%)	19 (37.3%)	
Primary differentiation			
Well to moderate	63 (90.0%)	43 (84.3%)	.349
Poor	7 (10.0%)	8 (15.7%)	
Primary pT stage			
1/2	11 (15.7%)	17 (33.3%)	.023*
3/4	59 (84.3%)	34 (66.7%)	
Primary pN stage			
0	44 (62.9%)	28 (54.9%)	.454
1/2	26 (37.1%)	23 (45.1%)	
Liver metastases			
No	46 (65.7%)	47 (92.2%)	.001**
Yes	24 (34.3%)	4 (7.8%)	

The median follow‐up time for all patients was 51.0 months (2‐82 months). OS of patients with high HDAC2 expression was significantly worse than that of patients with low expression (HR = 2.283, 95% CI =[1.003‐5.198], *P* = .031). For patients with high HDAC2 expression, the 3‐ and 5‐year OS rates were 76.1% and 58.3%, respectively. For patients with low HDAC2 expression, they were 87.3% and 74.6%, respectively (Figure 1C). These results suggested that higher HDAC2 expression was associated with poorer OS. *P < 0.05, **P<0.01, statistically significant.

### 3.2 *HDAC2 down‐regulation inhibited tumour cell migration and invasion via EMT* in vitro *and*in vivo

**FIGURE 2:**
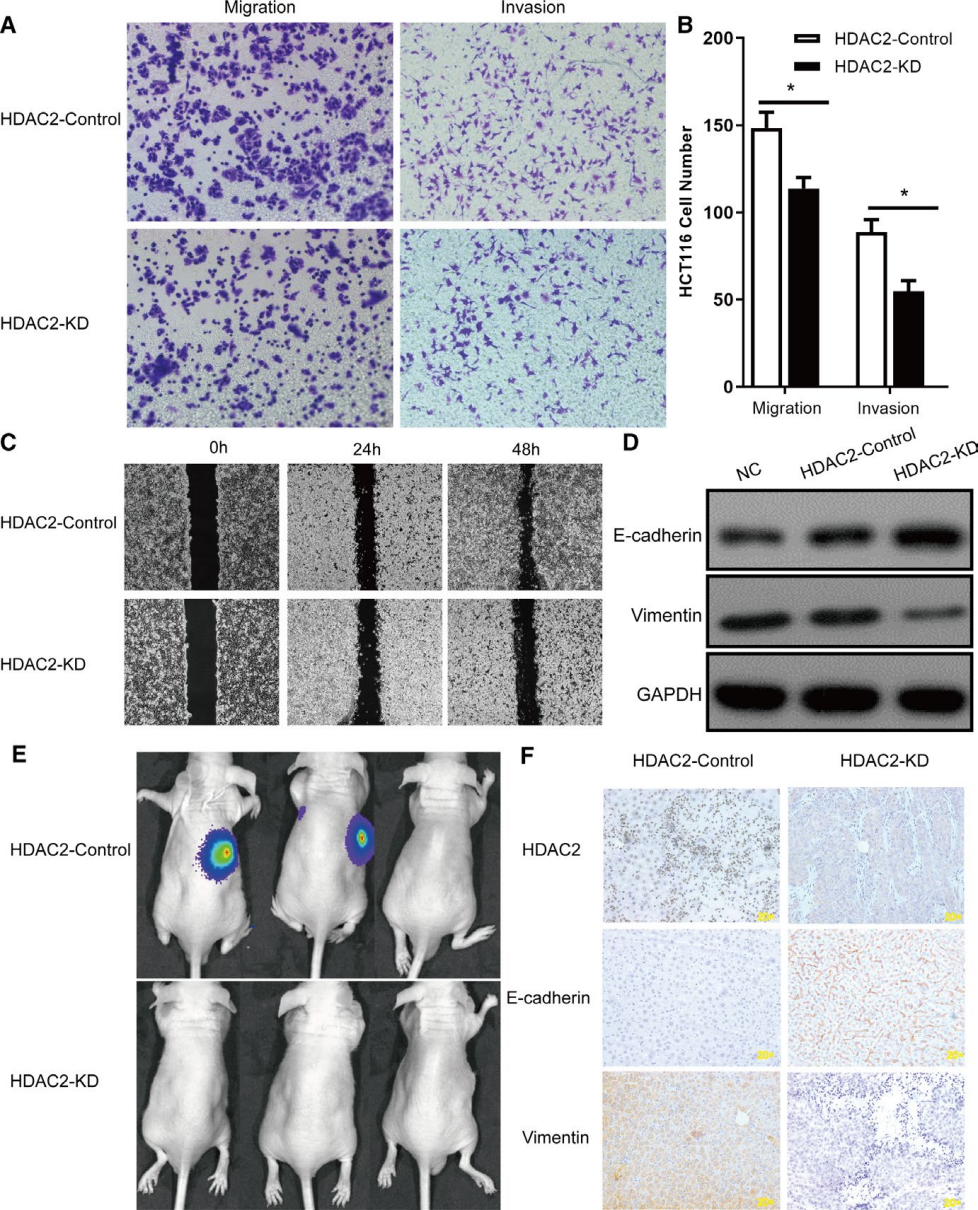
HDAC2 down‐regulation inhibited tumour cell migration and invasion via EMT in vitro and in vivo. (A) Migration and invasion for HDAC2‐Control cells and HDAC2‐KD cells of HCT116 and (B) Colony counts * *P* < 0.05. (C) Effect of HDAC2 on wound healing ability of HCT116. (D) Following HDAC2‐Control cells and HDAC2‐KD cells of HCT116, EMT genes were analysed by Western blotting. (E) When HDAC2 down‐regulation, the experimental group mice had significantly fewer liver metastatic tumours. (F) The expression levels of E‐cadherin, Vimentin and HDAC2 in liver tissues were measured by immunohistochemical analysis

HCT116 cells were infected with lentivirus containing either shRNA targeting HDAC2 or control scrambled shRNA. Western blotting and real‐time PCR analysis showed that HDAC2 effectively decreased both HDAC2 protein and mRNA levels (Figure S1). In transwell‐migration model, the results of 10 × microscopic observation showed that the number of transmembrane of HCT116 cells decreased significantly after HDAC2 down‐regulation (148.3 vs. 113.7, *P* = .0347). In the transwell‐invasion model, it gets the same result (88.7 vs. 54.7, *P* = .0229) (Figure 2A‐B) . All wound healing images showed that HDAC2 down‐regulation suppressed the cell migration ability and this effect was obviously concentration‐ and time‐dependent (Figure 2C). To address whether HDAC2 modulates tumour cell migration and invasion, the levels of the EMT marker, Vimentin and E‐cadherin, were detected. When HDAC2 down‐regulation, the expression of Vimentin was up‐regulated, whereas the expression of E‐cadherin was down‐regulated (Figure 2D).

HCT116 cells stably expressing control or HDAC2 shRNA were injected into the splenic capsule of nude mice to form xenograft tumours. HDAC2 down‐regulation reduced the tumour growth rate (Figure 2E). In addition, the expression of Vimentin was up‐regulated, whereas the expression of E‐cadherin was down‐regulated in tumours infected with Lv‐shHDAC2 as detected by IHC (Figure 2F). Taken together, these results suggest that HDAC2 may be a tumour metastasis‐promoting oncogene, and this process was closely connected with EMT.

### 3.3 ENSG00000274093.1 was binding to HDAC2 and was highly expressed in colorectal cancer tumour tissues

**FIGURE 3:**
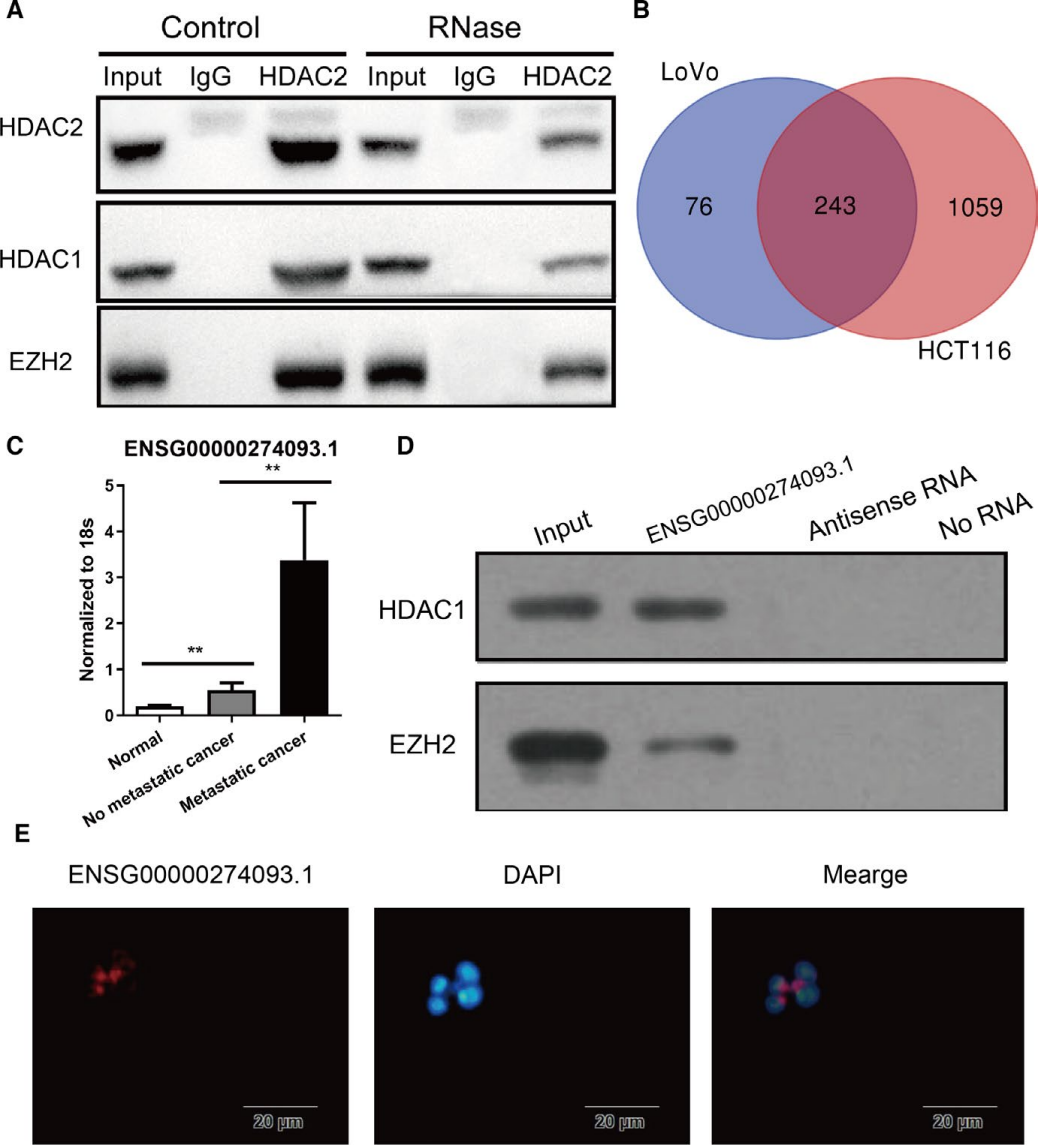
ENSG00000274093.1 was binding to HDAC2 and was highly expressed in colorectal cancer tumour tissues. (A) Co‐Immunoprecipitation experiments for the binding of HDAC2/ HDAC1 and EZH2 in RNase treatment and control HCT116 cells. (B) The Venn diagram shows the LncRNA binging with HDAC2 in LoVo cell (319 LncRNA), HCT116 cell (1302 LncRNA) or both (243 LncRNA). (C) Statistical analysis of lncRNA ENSG00000274093.1 expression in normal colorectal tissues, primary tumour tissues without liver metastasis and primary tumour tissues with liver metastasis. **<0.01. (D) Western blot of the proteins from antisense ENSG00000274093.1 and ENSG00000274093.1 pull‐down assays. (E) The localization of ENSG00000274093.1 in cells was detected by fluorescence in situ hybridization

For the mechanism of HDAC2 affecting EMT, Burstin et al found that HDAC2 bind to HDAC1 and then bind to Snail to up‐regulate the expression of E‐cadherin, thus promoting the occurrence of EMT in tumour cells.^16^ Subsequently, Tong et al found that EZH2 was also indispensable in the process of HDAC2/HDAC1/Snail complex affecting the expression of E‐cadherin. And HDAC2/HDAC1 complex plays a bridge role in the binding process of EZH2 and Snail.^17^ Moreover, small molecules could play a significant role in the process of the function of HDACs.^18^, ^19^, ^20^ Therefore, we evaluated whether RNA was needful for HDAC2/HDAC1 and EZH2 binding. Co‐Immunoprecipitation experiments showed that the binding of HDAC2/ HDAC1 and EZH2 decreased after RNase treatment in HCT116 cells (Figure 3A).

To determine the crucial RNA, especially LncRNA, an HDAC2‐RIP‐seq experiment was conducted. After analysis, the top 20 enrichment lncRNAs were selected (Figure 3B). Next, tissue samples from 30 patients were used to screen these 20 lncRNAs. It was found that the expression levels of 3 lncRNAs (ENSG00000274093.1, ENSG00000280408.1, ENSG00000259715.1) in normal colorectal tissues, primary tumour tissues without liver metastasis and primary tumour tissues with liver metastasis were gradually increased, which may be consistent to the progress of colorectal cancer (Figure 3C & Figure S2). And lncRNAs pull‐down showed that HDAC1 and EZH2 could bind to ENSG00000274093.1, rather than ENSG00000280408.1, ENSG00000259715.1 (Figure 3D).

LncRNA ENSG00000274093.1, located on chr16:69632141‐69632571:1, is a kind of sense intronic lncRNA. The ENSG00000274093.1 has its copy from the introns of NFAT5 that does not overlap any exons.( Figure S3) The distribution of lncRNAs in cells is different, and the function mechanism is also different.^11^ We found that ENSG00000274093.1 was mainly located in the cell nucleus (Figure 3E). This suggests that ENSG00000274093.1 may play a role in regulating the binding of HDAC2, HDAC1 and EZH2.

### 3.4 ENSG00000274093.1 down‐regulation inhibited HDAC1/HDAC2 and EZH2 binding and tumour cell EMT

**FIGURE 4:**
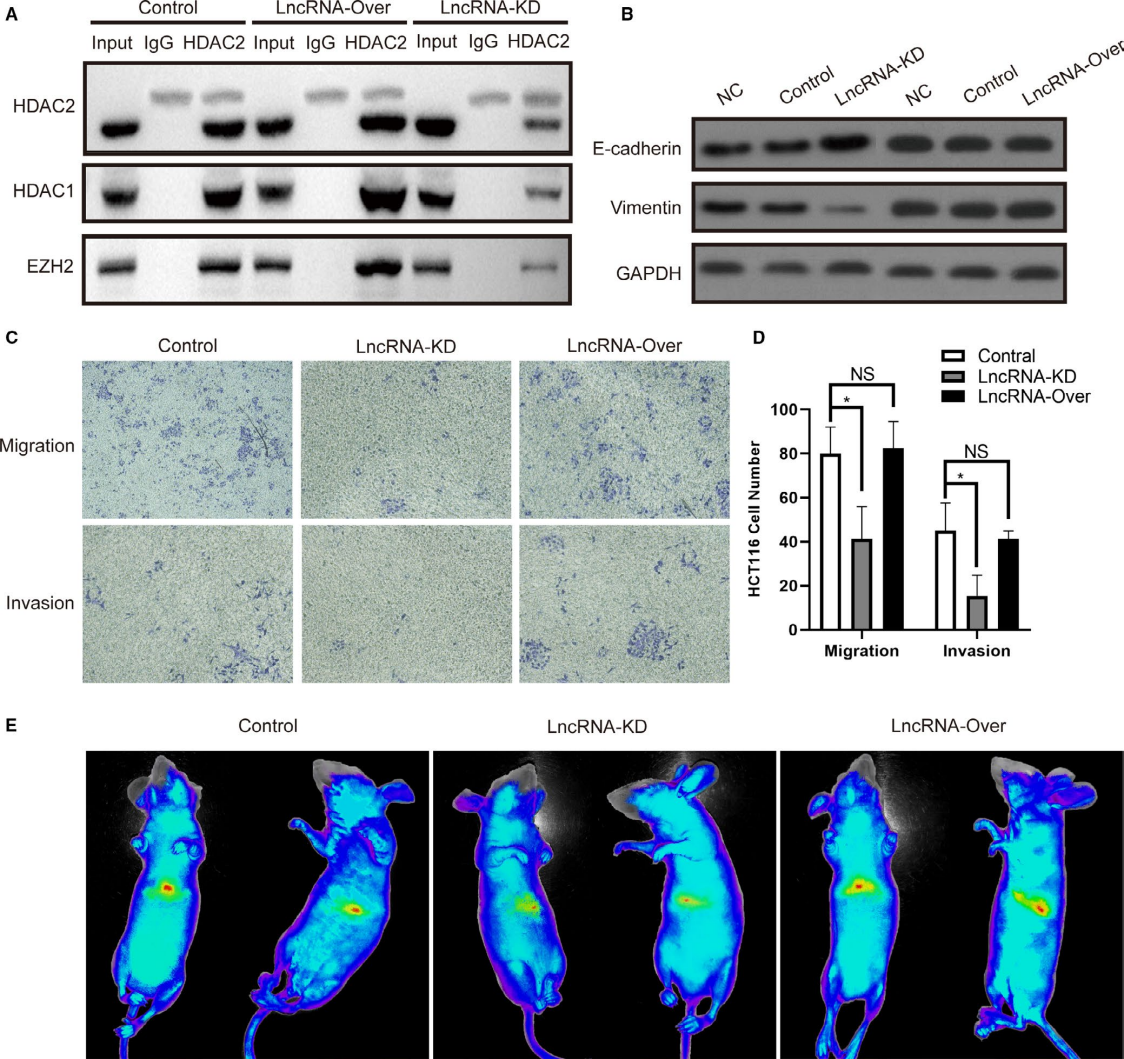
ENSG00000274093.1 down‐regulation inhibited HDAC1/HDAC2 and EZH2 binding and tumour cell EMT. (A) Co‐Immunoprecipitation experiments for the binding of HDAC2/ HDAC1 and EZH2 in ENSG00000274093.1‐Over cells and ENSG00000274093.1‐KD cells of HCT116. (B) Western blot of the EMT proteins in ENSG00000274093.1‐Over cells and ENSG00000274093.1‐KD cells of HCT116. (C) Migration and invasion for ENSG00000274093.1‐ KD cells and ENSG00000274093.1‐Over cells of HCT116 and (D) Colony counts *P < 0.05. (E) The metastatic tumour formation rate decreased significantly after ENSG00000274093.1 down‐regulation. There was no significant change in the metastatic tumour formation rate after overexpression of ENSG00000274093.1

To verify the function of ENSG00000274093.1, we conducted the Co‐Immunoprecipitation experiments. The results showed that after ENSG00000274093.1 overexpression (Figure S4), the binding of HDAC1/HDAC2 and EZH2 did not change, whereas after ENSG00000274093.1 down‐regulation, the binding of HDAC1/HDAC2 and EZH2 decreased (Figure 4A). Moreover, when ENSG00000274093.1 down‐regulation, the expression of E‐cadherin was up‐regulated, whereas the expression of Vimentin was down‐regulated. And there was no significant change of Vimentin and E‐cadherin when ENSG00000274093.1 overexpression (Figure 4B).

In transwell‐migration model, the results showed that the transmembrane number of HCT116 cells decreased significantly after ENSG00000274093.1 down‐regulation (80.0 vs.41.3, *P* = .0238). There was no significant change in the number of HCT116 cells after overexpression of ENSG00000274093.1 (80.0 vs. 82.4, *P* = .8250). In transwell‐invasion model, it showed similar results (Figure 4C‐D). The animal experiment showed similar results to the cell test; the metastatic tumour formation rate decreased significantly after ENSG00000274093.1 down‐regulation. There was no significant change in the metastatic tumour formation rate after overexpression of ENSG00000274093.1 (Figure 4E).

## 4 DISCUSSION

In this study, we found that HDAC2 expression is increased in CRC with liver metastasis as compared to CRC without liver metastasis and adjacent normal tissues. Clinicopathologic data showed that increased HDAC2 expression is correlated with liver metastasis and higher T stages. Moreover, patients with higher HDAC2 expression had reduced OS. This indicated that abnormal HDAC2 expression might be associated with liver metastasis of CRC. To explore the function of HDAC2 in CRC, we knocked down HDAC2 in HCT116 cells via shRNA. The results showed that HDAC2 down‐regulation decreased HCT116 cell migration and invasion via the EMT pathway. Besides, HDAC2 knockdown suppressed CRC cell liver metastasis in nude mouse xenografts.

Previous studies show that HDACs plays a key role in the regulation of EMT in a variety of tumours.^21^, ^22^ A main mechanism is that Snail can recruit HDAC1/2 and Sin3A complexes into the E‐cadherin promoter, then down‐regulate the expression of E‐cadherin, and promote the metastasis of pancreatic cancer.^16^ Also, further studies found that the role of Snail/HDAC1/HDAC2 complex needs to be mediated by EZH2.^17^ In addition, it has been found that E‐box binding ZEB1 can also regulate the recruitment of HDAC2 to the E‐cadherin promoter region. However, some details are still unclear. Some small moleculars, such as RNA, may act as chaperones when HDACs work. We hypothesized that HDAC2 knockdown‐mediated liver metastasis of CRC may need RNA. Co‐Immunoprecipitation experiments showed that, compared with the control group, the binding of HDAC1/ HDAC2 and EZH2 decreased after RNase treatment in HCT116 cells (Figure 1). This suggests that RNA was necessary for HDAC1/HDAC2 and EZH2 binding.

In recent years, there have been more and more studies on the role of LncRNA as an important molecule involved in the role of histone modification and other biological processes of cancer.^23^, ^24^, ^25^, ^26^ Such as LncRNA‐HOTAIR can act as a modular scaffold for the binding PRC2 complex and LSD1/CoREST/REST, and then affect the methylation level of histone H3K27.^27^ And it also can form a Snail/HOTAIR/EZH2 trimer complex with EZH2 and Snail, and then affect EMT.^28^ However, more specific mechanisms of lncRNAs andEMT need to be further teased out. So, we examined lncRNA expression profiles in HDAC2‐RIP, from which we identified and characterized a novel lncRNA, ENSG00000274093.1, which is highly expressed in colorectal cancer tumour tissues. Mechanistically, lncRNAs pull‐down revealed that ENSG00000274093.1 not only modified genomic binding of HDAC2, but also HDAC1 and EZH2. Furthermore, ENSG00000274093.1 down‐regulation inhibited tumour cell migration and invasion. However, there was no significant change in the number of HCT116 cells after overexpression of ENSG00000274093.1. Those results strongly suggest that ENSG00000274093.1 may act as a scaffold for HDAC2/HDAC1/EZH2, and then promotes tumour cell EMT and cell migration and invasion. Thus, our study has identified a novel LncRNA, ENSG00000274093.1, which bound HDAC2, HDAC1 and EZH2, and may act as a modular scaffold of HDAC1/HDAC2 and EZH2 Complexes, and consequently altered EMT of the colorectal cancer cell.

Given the clinical, biochemical and functional significance of HDAC2 in CRC, we conclude that HDAC2 and its associated LncRNA, ENSG00000274093.1, are crucial for liver metastasis of CRC, and targeting this pathway may be pivotal in the prevention or treatment for liver metastasis of CRC.

## CONFLICTS OF INTEREST

The authors declare no conflict of interest.

## AUTHORS CONTRIBUTIONS

Zhipeng Qi: Conceptualization (equal); Investigation (equal); Project administration (equal); Writing‐original draft (lead); Writing‐review & editing (lead). Ayimukedisi Yalikong: Investigation (equal). Jiawei Zhang: Investigation (equal). Shilun Cai: Methodology (equal). Bing Li: Software (equal). Di Sun: Data curation (equal). Zhentao Lv: Methodology (equal). Enpan Xu: Visualization (equal). Yunshi Zhong: Conceptualization (equal); Funding acquisition (lead); Project administration (equal); Supervision (equal). Pinghong Zhou: Funding acquisition (equal); Supervision (equal).

## Supporting information

Supplementary Material
