# Supplementary material for: HDAC2 promotes the EMT of colorectal cancer cells and via the modular scaffold function of ENSG00000274093.1
Source: J Cell Mol Med. 2020 Dec 15;25(2):1190–7. doi: 10.1111/jcmm.16186 (PMC7812252; doi:10.1111/jcmm.16186)
Supplement: Supplementary file 1 — Supplementary Material [file JCMM-25-1190-s001.pdf]

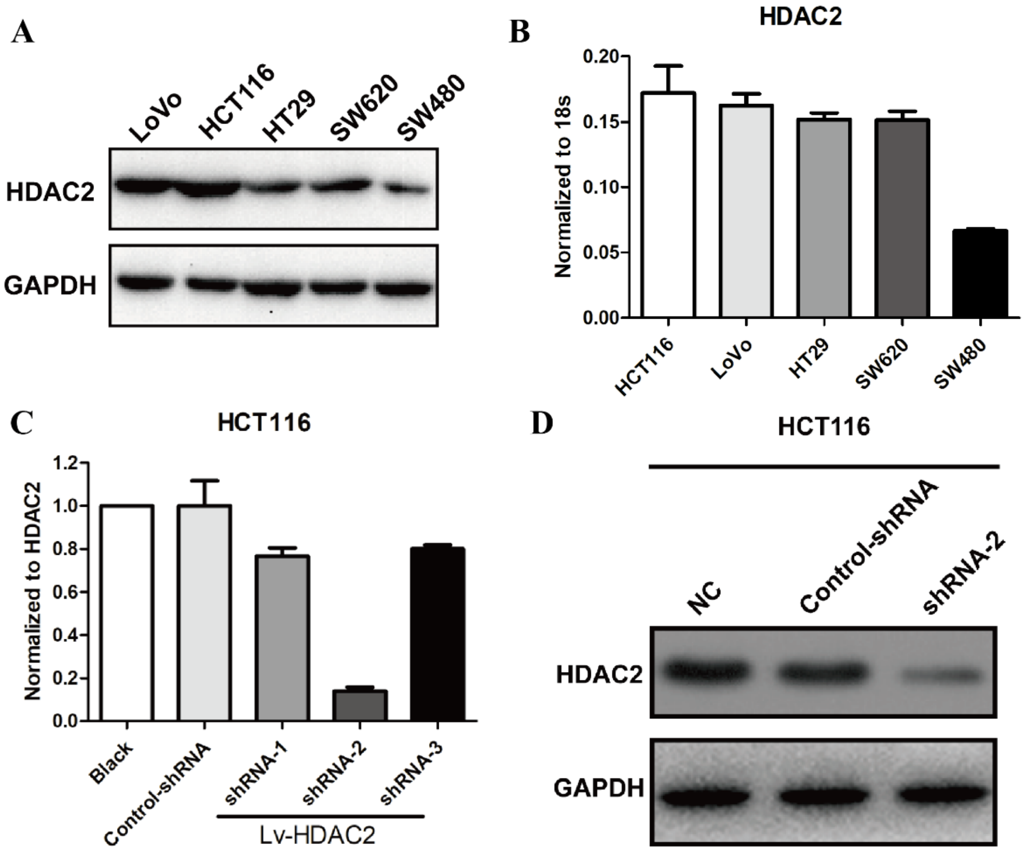

Figure S1: HDAC2 was downregulated in HCT116. (A-B) Western blotting analysis for HDAC2 in the usually used human colorectal cancer cell lines. (C) RT-PCR analysis for HDAC2 mRNA levels in untreated (Black) and Lv-Control-shRNA- or Lv-shRNA 1-3-infected HCT116 cells. Western blotting analysis for HOXC6 in untreated (NC) and Lv-Control-shRNA- or Lv-shRNA2 -infected HCT116 cells, with GAPDH as the control.

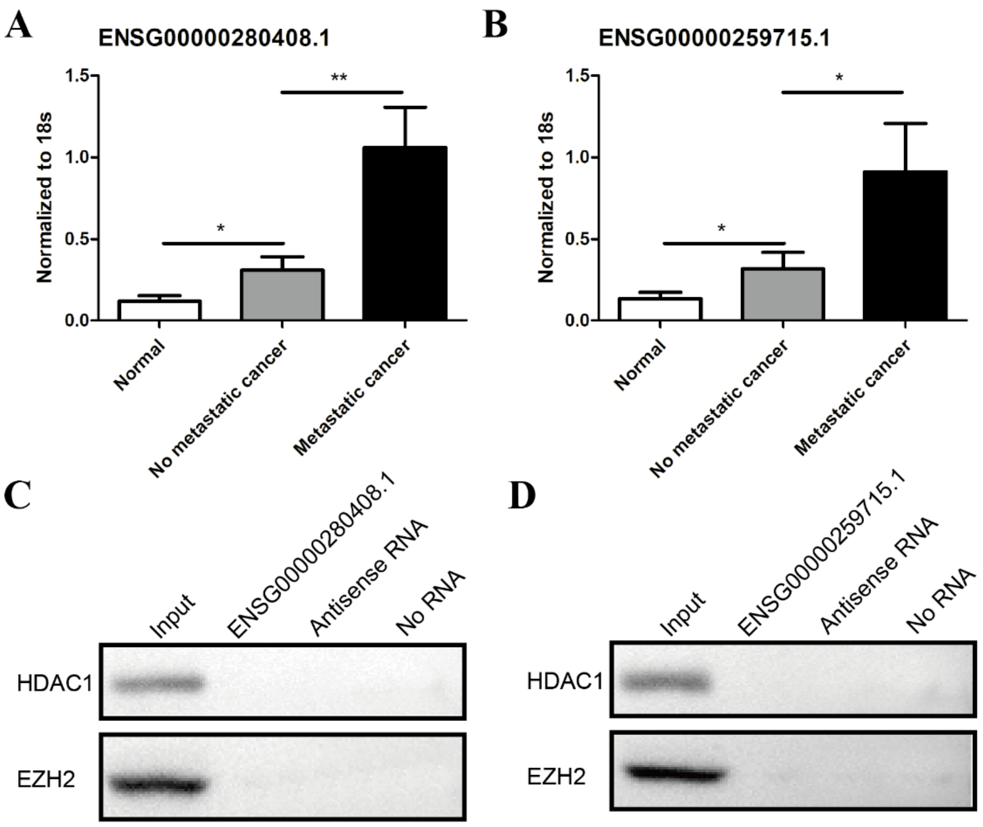

Figure S2: lncRNA ENSG00000280408.1, ENSG00000259715.1 was high expression in colorectal cancer tumor tissues, but not binding to HDAC1 and EZH2. (A-B) ENSG00000280408.1, ENSG00000259715.1 expression in in normal colorectal tissues, primary tumor tissues without liver metastasis and primary tumor tissues with liver metastasis. (C) Western blot of the proteins from ENSG00000280408.1, ENSG00000259715.1 pull-down assays.

A

LncRNA ENSG00000274093.1 sequence

TGAATTAAAATCACAGAAATTTCTAAACCATCTATAATAAT
ACACTGTACAAAAACTGAGGTGAAGAAAATATGTTACTC
CTCCTGTAGGAGACAGCTTTTAAAAAATGAAAATATATTA
TCACTTTTAAGTTCCTACTATGTGACAAGAATTGTGTTAG
GTAATTTGCATGTCATCATATCACAATCCTTCTAGCCATA
AAGTATCTCTCTTTTTTCTTTTACCTAGTTCTTACTCCTT
GTCATTGTGCTTGAGGGAATTTGGCATTGTGTGCTATCA
CTTCGATTAGGGAAACGCAAGATCCTTAACTAGGCAATG
TATAGAATATTGAGTTGAAAAGCCATACCTCTTCTCTACA
AGTGTCAGGATCTTCGAATGGGAATTTGATTTCTATAATA
ATAATTTAATAAAAGTGATTTCTAGCAACAAAA

B

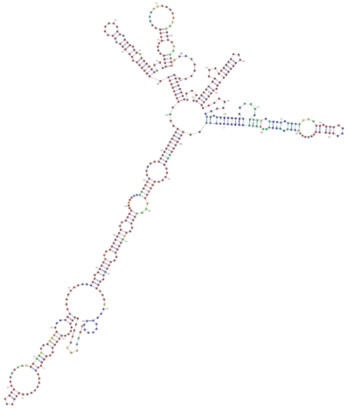

Figure S3: The details of lncRNA ENSG00000274093.1. (A) The full length Sequence of ENSG00000274093.1. (B) the RNA secondary structure of this LncRNA was predict in the NONCODE database (http://www.noncode.org/).

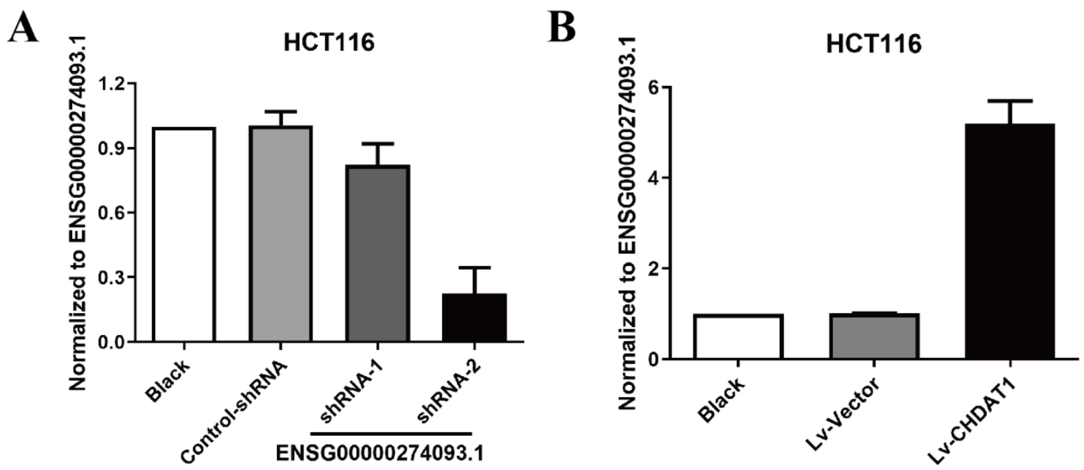

Figure S4: ENSG00000274093.1 was downregulated and upregulated in HCT116. (A) RT-PCR analysis for ENSG00000274093.1 levels in untreated (Black) and Lv-Control-shRNA- or Lv-shRNA 1-2-infected HCT116 cells. (B) RT-PCR analysis for ENSG00000274093.1 levels in untreated (Black) and Lv-Vector-or ENSG00000274093.1-infected HCT116 cells.

Table S1: The Primer of the 20 LncRNA

	Forward Primer	Reverse Primer
ENSG00000264558.1	TTGTTTTTCTCTTTTCC AAGATGGC	CATCTTTTGAAAAACCT GGAAGTGA
ENSG00000279541.1	ATAAAAAATGCTGCTG AGAACATGG	ATGGGGTCTTGCTGTGT TATTCA
ENSG00000274630.1	ATGAAGCTTCTTTGTG TGGACTGAA	CAGTGGAGCCTGCTTTA T
ENSG00000249731.1	ATGATGCTGTTGTGGC TGTTGG	ATGGCAACCAGCTGTTC TGC
ENSG00000228830.1	TTCACTTTCACATCTC CAGCAAGG	GCTAGTGGAGAGGTCTA TTGTCCCA
ENSG00000279337.1	AGCACTTTGGGAGGC AGAGG	GAGGCGGGGTTTCACTG TG
ENSG00000256512.1	AATGCTGTCCTTACCT TAGTCC	GGAAGGGAAGAAAACA CCA
ENSG00000260257.2	TGACCTTGTGATCCAC CTGCC	TGGCAGTAGGTTGAGGT CAGTAACT

ENSG00000272579.1	CAGGGTCCTTTCCTTGGAAATGAA	CACTTTGCCTTTCAAAGCAGGAGT
ENSG00000257181.1	ACCCATTAAAAGTGGTTTTGTGTGC	TCTCAGTGCCTTTTGCAATTTGT
ENSG00000275413.1	TGATGTTAACATCACGCGTAGGCA	CGTTGCCACAAAATATCTCGCT
ENSG00000256364.1	AGAACTCTCAGCCCAGCTCTGC	CCAGCTGACTGAAAACAAGGACAG
ENSG00000274093.1	TACTCCTTGTCATTGTGCTTGAGG	CCATTCGAAGATCCTGACACTTGT
ENSG00000253744.1	ACAAGTCCCGGAGGCACAGA	CGCCGTCCTGCTCTCCTTA
ENSG00000259715.1	TATGCTTTGCACAGTGGCTGG	GCTCATTTTCTCCCTCCATGTAGAC
ENSG00000280408.1	ACTGCAACCGGCATAGGACAGAT	TTTCAGGCAGCTACTCCCAGC
ENSG00000279331.1	ACCAAAGGAATGACCAATGAGAGC	GGATGATTCCCGTTTGAGGATGT
ENSG00000273568.1	TTGTCCACAACTCCCAGGGG	CGGTCCAGGTTAATTTGAAGGC
ENSG00000280320.1	TAGAATATAAGGCCCCTCTGGAACC	GCATGTGCAATAGAAGCCTTCCTT
ENSG00000263786.1	AGTCTGGGTGACAGAGTGAGACCTT	TGGCAACAGAGAGACACCTGGT
